# News and Notes

**Published:** 1902-01

**Authors:** 


### NEWS AND NOTES.

THE *Atlanta Medical and Surgical Journal* and *Southern Medical Record* having been consolidated into the ATLANTA JOURNAL-RECORD OF MEDICINE, exchanges will please be sent only to the latter. We are getting many duplicates.

THE segregation of lepers in the Island of Bary, Philippine Islands, has been recommended.

THE physicians of Montreal are making great efforts to establish a sanitarium for tuberculosis patients near that city.

WILLIAM WALDORF ASTOR has presented $50,000 to the National Society for the Prevention of Cruelty to Children.

IN the town of Juan Diaz, Porto Rico, there are 2,500 inhabitants, 1,000 of which are estimated to be victims of the morphine habit.

$10,000 has been donated by Dr. Kelly to Johns Hopkins Hospital; the money will be used to build a large addition to the gynecological department.

THE nurses of Boston are taking steps to establish the McKinley Order of Nurses, to be in this country what the Victoria Order of Nurses is in England.

A PROJECT to build a hospital as a memorial to the late Dr. A. J. C. Skene has been abandoned; the money which was subscribed has been returned to the contributors.

SURGEON HARRIS, of the U. S. cruiser Albany, in a report to the surgeon-general of the navy with regard to the condition of his ship, says that from a sanitary point of view the Albany is so radically wrong as regards construction that it is almost impossible to relate in detail the necessary changes which will have to be made before this ship will become a sanitary vessel.

---

THE Canadian Director-General of Public Health has issued a circular to all Canadian steamship companies requesting medical officers of vessels to prepare and hand to the Canadian health officers on the arrival at Canadian ports a statement of the temperature of every person on board taken within 24 hours of arrival. This measure has been adopted in order to guard against plague.

---

DAVID HARRIS, former health officer on the diamond fields, South Africa, writes the *Lancet* that he practiced in that region in the early "eighties," when malaria was very prevalent there, and he avers the entire absence of mosquitoes. The population numbered about 70,000. With extension of the railway from Cape Town 2 or 3 years later came the first installment of mosquitoes. But malaria steadily decreased in his own very large practice and elsewhere in general.

---

THE *Army and Navy Register* records that an alarming percentage of army horses in Cuba have gone stone blind. Of the 2,808 animals inspected in the course of a year, 694, or almost 25 per cent., were condemned for blindness. Of the 694 horses thus afflicted 484 were in Cuba. The affliction does not yield to treatment and the veterinarians are at a loss to explain or account for it, though they have concluded that it is not caused by the glare and heat of the tropic sun, as it was at first supposed. In the Philippines, where the climate is much the same as in Cuba and Porto Rico, no such disease appears to exist so far as indicated by the figures. In these latter islands a great majority of the animals condemned during the year had glanders, but it is reported the disease is now being gotten under control so that its spread may be largely prevented.

---

THE new Manila Medical Society is meeting, says the *Journal of the American Medical Association*, with opposition from the Collegio de Medicosy Pharmaceuticos. The members of that school claim that no new medical society is needed. There is a so-called society of physicians and pharmacists already in existence, but it is claimed that there have been but two or three meetings of the body within two years, and then only for the purpose of discussing politics, and not questions of importance to the profession, with which the members of the society are supposed to be identified. It will be readily seen that a new medical organization is sadly needed. It is the intention of the new society to hold semi-monthly meetings for the purpose of discussing questions pertaining to the practice of medicine and surgery. As a well known army surgeon remarked: "When a Filipino graduates his studies are over. He does not keep abreast of the times. The physicians of Manila, outside of the Americans and one or two whom I have in mind, are many generations behind the times. They know practically nothing whatever regarding the modern practice of medicine and surgery. They have taken no steps to investigate the diseases of the tropics; especially has this been the case in regard to dysentery. Very little is known regarding amebic dysentery, and what little is known has been discovered by American practitioners. If the local doctors had made a study of this disease ten years ago, there is no doubt that we would know how to cope with it successfully by this time." The new society will publish a monthly medical journal, in which will appear the proceedings of the society at the various meetings, as well as original articles by the members of the society.

AN interesting paper, says the *British Medical Journal*, was read at the Pan-Hellenic Medical Congress recently held at Athens. Mr. P. Kavadias, Inspector-General of Antiquities in Greece, combated the view commonly held that the temples of Æsculapius at Epidaurus, in Asia Minor, in Crete, and at Rome, were schools of medicine as well as shrines. In opposition to all writers, archeological and medical, who have dealt with the subject, he maintained that the Asclepieia were not hospitals, and the priests were not in any sense physicians. By the records of cures inscribed on the walls of the Temple of Epidaurus he disproved the statements of Strabo and of Pliny that Hippocrates founded his teaching partly on cures wrought in the Temples of Æsculapius, at Cos, and even copied all the therapeutic formulæ among the inscriptions in that temple. M. Kavadias contended that the Epidaurus inscriptions themselves conclusively show that no medical art was practiced in the temples of Æsculapius in the time of Hippocrates. He argued that medicine was originally a folklore, the development of which was altogether independent of religion. The first physicians were called Asclepiadæ; but, so far from being priests in the temples of Æsculapius, they were empiric practitioners who learnt their art by tradition, secrets of healing being handed down in the family. In the time of the Roman diminion, however, and especially after the beginning of the Christian era, a great change took place. The temples of Æsculapius, while preserving their religious character, gradually became transformed into sanatoria in which sufferers were treated by the priests, but in the light of such medical science as was available. Patients were persuaded by various artifices that the god Æsculapius himself prescribed the treatment, to which they were expected to submit with religious faithfulness. The sick folk slept in the temples, and their dreams were interpreted for them in an appropriate manner by the priests. The hierophantic part of the procedure was the use of what would nowadays be called suggestion, whereby the patients were induced to believe that the treatment was dictated by the god himself under symbols and images. The system had evidently the great advantage of making the patient submissive, and modern practitioners might sometimes be tempted to envy the "metaphysical aid" which the priest-physicians of Epidaurus in its later days could call to their aid.

